# Effects of Resveratrol in Goto-Kakizaki Rat, a Model of Type 2 Diabetes

**DOI:** 10.3390/nu11102488

**Published:** 2019-10-16

**Authors:** Katarzyna Szkudelska, Marzanna Deniziak, Iwona Hertig, Tatiana Wojciechowicz, Marianna Tyczewska, Magdalena Jaroszewska, Tomasz Szkudelski

**Affiliations:** 1 Department of Animal Physiology and Biochemistry, Poznan University of Life Sciences, Wołyńska 35, 60-637 Poznań, Poland; katarzyna.szkudelska@up.poznan.pl (K.S.); iwona.hertig@up.poznan.pl (I.H.); tatianag@up.poznan.pl (T.W.); magdalena.j1@o2.pl (M.J.); 2 Department of Biochemistry and Cell Biology, University of Rzeszow, Zelwerowicza 4, 35-601 Rzeszów, Poland; deniziak@univ.rzeszow.pl; 3 Department of Histology and Embryology, Poznan University of Medical Sciences, Święcickiego 6, 60-781 Poznań, Poland; maritycz@ump.edu.pl

**Keywords:** resveratrol, type 2 diabetes, Goto-Kakizaki rats

## Abstract

Resveratrol exhibits a pleiotropic, favorable action under various pathological conditions, including type 2 diabetes. However, its anti-diabetic effects in animal models and human trials have not been fully elucidated. The aim of the present study was to determine whether resveratrol is capable of inducing beneficial changes in the Goto-Kakizaki rat, a spontaneous model of diabetes, which in several aspects is similar to type 2 diabetes in humans. Goto-Kakizaki (GK) rats and control Sprague–Dawley (SD) rats were treated intragastrically with resveratrol (20 mg/kg b.w./day) for 10 weeks. Then, a glucose tolerance test was performed and levels of some adipokines in blood were measured. Moreover, lipid contents in skeletal muscle and liver tissues, along with the expression and phosphorylation of pivotal enzymes (AMP—activated protein kinase—AMPK, acetyl-CoA carboxylase—ACC, protein kinase B—Akt) in these tissues were determined. Histology of pancreatic islets was also compared. GK rats non-treated with resveratrol displayed a marked glucose intolerance and had increased lipid accumulation in the skeletal muscle. Moreover, upregulation of the expression and phosphorylation of AMPK, ACC and Akt was shown in the muscle tissue of GK rats. Those rats also had an abnormal structure of pancreatic islets compared with control animals. However, treatment with resveratrol improved glucose tolerance and prevented lipid accumulation in the skeletal muscle of GK rats. This effect was associated with a substantial normalization of expression and phosphorylation of ACC and Akt. In GK rats subjected to resveratrol therapy, the structure of pancreatic islets was also clearly improved. Moreover, blood adiponectin and leptin levels were partially normalized by resveratrol in GK rats. It was revealed that resveratrol ameliorates key symptoms of diabetes in GK rats. This compound improved glucose tolerance, which was largely linked to beneficial changes in skeletal muscle. Resveratrol also positively affected pancreatic islets. Our new findings show that resveratrol has therapeutic potential in GK rats.

## 1. Introduction

Resveratrol (3,5,4′-trihydroxystilbene) is a naturally-occurring diphenolic compound present in different plant species. It belongs to a biologically active agents raising huge interest due to its pleiotropic, health-promoting effects that have been shown under various pathological conditions. Resveratrol is also present in red wine and is thought to be one of the key polyphenols responsible for the so-called “French paradox”, i.e., a reduced incidence of cardiovascular disease in humans as a result of moderate consumption of red wine [1]. Resveratrol was demonstrated to exert, among others, anti-cancer [2], cardio-protective [3], neuro-protective [4], anti-oxidative [5], anti-obesity [6] and anti-inflammatory [7] effects. Moreover, its action in ameliorating non-alcoholic fatty liver disease [8] and ethanol-induced metabolic disturbances [9,10] has been relatively well documented. Resveratrol was also reported to promote mitochondrial biogenesis and to increase survival of animals exposed to a high-fat diet [11]. It is believed that this compound positively affects metabolism and energy expenditure, thereby reducing adiposity and adiposity-related metabolic disorders [6].

The intracellular action of resveratrol involves modulation of key signaling molecules, including AMP-activated protein kinase (AMPK) and silent information regulator 1 (SIRT1). AMPK is an important energy sensor implicated in the regulation of several physiological processes, such as energy metabolism as well as mitochondrial function. Moreover, AMPK is known to markedly influence the action of hormones regulating metabolic processes, especially insulin and adiponectin. SIRT1 is an NAD^+^-dependent histone deacetylase associated with the regulation of mitochondrial biogenesis, inflammation, intracellular metabolism, stress resistance, apoptosis and glucose homeostasis [6].

Apart from these effects, there is a large body of evidence showing that resveratrol also has a considerable anti-diabetic potential [12]. Studies with animal models indicate that this compound is capable of ameliorating many symptoms of diabetes, while also attenuating diabetes complications. However, results of human trials addressing its anti-diabetic action are less conclusive. Some studies demonstrated that resveratrol induces beneficial effects in humans with type 2 diabetes [13,14], whereas other authors indicated a lack of such effects [15,16]. Anti-diabetic properties of resveratrol are very intriguing, given the high prevalence of diabetes worldwide and still increasing morbidity. Pharmacological treatment of diabetes is very effective; however, it is accompanied by some side-effects. Therefore, various natural compounds, including resveratrol, are continually being tested to support conventional therapies as well as minimizing the side-effects of anti-diabetic drugs. In this context, resveratrol was demonstrated to be well tolerated for long periods [17]. However, its mechanism of action should be fully elucidated, especially using the best available animal models of diabetes. Among various rodent models of type 2 diabetes, Goto-Kakizaki (GK) rats are thought to be one of the best. These animals are non-obese and develop diabetes spontaneously. Given that in humans with a genetic predisposition to type 2 diabetes, the onset of the disease may be influenced by some environmental factors (such as feeding or physical activity), the utility of this model in research is relatively high. GK rats differ from other rodent models of type 2 diabetes, in which insulin resistance develops as a result of dietary intervention [18,19]. Importantly, many disorders appearing in this model are comparable to changes observed in humans with type 2 diabetes. GK rats display the key hallmarks of type 2 diabetes, i.e., mild hyperglycemia, insulin resistance and a progressive failure of pancreatic β-cells [18,19,20]. Moreover, both some anti-diabetic drugs and naturally-occurring agents were shown to exert a beneficial action in GK rats [18,21]. However, the efficacy of resveratrol in this model had not been examined. Although resveratrol action is relatively well explored in rodent models with diet-induced insulin resistance, the etiology of diabetes in GK rats is different. It is also known, that resveratrol action is tissue specific [6,7,12]. Moreover, in various models of diabetes, including GK rats, metabolic dysfunction in tissues is not identical [18,19]. Therefore, resveratrol in GK rats may either be ineffective or its action may be completely different. The aim of the present study was to determine whether this compound is capable of ameliorating symptoms of diabetes in GK rats. Moreover, the mechanism of its action in this diabetes model has been proposed.

## 2. Materials and Methods

### 2.1. Animals and Treatment

Three-week-old male Goto-Kakizaki (GK) rats and age-matched male Sprague–Dawley (SD) rats (purchased from Taconic Biosciences Inc., Germantown, MD, USA) were housed in an air conditioned animal room with a 12/12 h dark-light cycle and a constant temperature 21 ± 1 °C. Animals had free access to tap water and were fed a diet recommended for GK rats (Rodent NIH-31 M Auto diet, Zeigler Bros. Inc., Gardners, PA, USA) containing 18% crude protein, 5% crude fat, 5% crude fiber and gross energy at 4.02 kcal/g. After a 3-week adaptation period, rats were randomly divided into 4 groups consisting of 10 animals each: GK rats receiving vehicle (GKC), GK rats receiving resveratrol (GKR), SD rats receiving vehicle (SDC) and SD rats receiving resveratrol (SDR). Animals were maintained in plastic cages with 2 or 3 individuals per cage. Resveratrol (from Cayman Chemicals, Ann Arbor, MI, USA) was dissolved in 0.5% carboxymethylcellulose (Sigma-Aldrich, St. Louis, MO, USA) and 10 mL of this solution (or vehicle without resveratrol) per kg b.w. were given intragastrically by gavage once a day for 10 weeks (from the 6th to 16th week of animal life). The volume of the solution administered to animals was chosen to obtain the most homogeneous suspension of resveratrol. The dose of resveratrol was 20 mg/kg b.w. During the entire experiment, body weight was checked twice a week. After 10 weeks of treatment, non-fasted rats were killed by decapitation and their tissues (blood, skeletal muscle—musculus biceps femoris, liver) were sampled and stored for analysis at −80 °C. Moreover, liver and pancreas scraps were stored in Bouin’s solution.

The experiment was performed with the permission (no. 48/2016; date of approval 1 July 2016) of the Local Ethical Commission for Investigations on Animals in Poznan according to the Act on the Protection of Animals Used for Scientific or Educational Purposes in Poland adopted on 15 January, 2015, which complies with current EU regulations.

### 2.2. Glucose Tolerance Test and Fasting Blood Insulin Levels

After 8 weeks of treatment with the resveratrol solution or vehicle alone, rats were fasted overnight (12 h) and the glucose tolerance test (GTT) was performed. To minimize stress related with fasting, GTT was performed only once during the experiment. Given that fasting and glucose treatment induce hormonal and metabolic changes, rats were subjected to the test two weeks before the end of the study. Thus, after 8 weeks of the experiment blood samples were collected from the tail vein and glycemia was measured using a glucometer (Diagnosis S.A., Białystok, Poland). This was followed by the intragastric administration of glucose solution (0.5 g/kg b.w., dissolved in 10 mL of water) and glycemia was measured 30, 60, 120 and 180 min after glucose loading. Additionally, blood samples were collected from the tail vein before glucose administration to determine fasting insulin levels. Insulin was measured by the rat hormone-specific radioimmunoassay test (Millipore, St. Charles, MO, USA).

### 2.3. Blood Adipokine and HbA1c Levels

Leptin levels in blood serum were determined by the radioimmunoassay test (multispecies, Millipore, St. Charles, MO, USA). Moreover, ELISA kits were used to determine a rat-specific adiponectin (R&D Systems Inc., Minneapolis, MN, USA), resistin, apelin and visfatin (Shanghai Sunred Biological Technology Co., Shanghai, China). Apart from adipokines, glycated hemoglobin (HbA1c) was assayed in blood using the rat hemoglobin A1c kit (Crystal Chem, Inc., Downers Grove, IL, USA). All these parameters were determined in non-fasted animals after 10 weeks of the experiment.

### 2.4. Liver and Muscle Lipids

Total lipids were extracted from skeletal muscle and liver tissue according to method of Folch et al. [22]. To determine triglycerides (TG), small amounts (50 µL) of tissue extracts were lyophilized and then triglycerides were assayed using Pointe Scientific enzymatic kits (Canton, MI, USA). Non-esterified fatty acids (NEFA) were measured in tissue extracts colorimetrically by the method of Duncombe [23]. Reagents needed for the determination of fatty acids were purchased from Sigma-Aldrich (St. Louis, MO, USA).

### 2.5. Western Blot Immunodetection

Total protein extracts of rat liver and skeletal muscle tissue were prepared using the T-PER Tissue Protein Extraction Reagent (Thermo Scientific, Waltham, MA, USA), with minor modifications of the recommended lysis procedure. Tissue (≈20 mg) was suspended in the appropriate amount of cold lysis buffer, supplemented with protease and phosphatase inhibitor (Thermo Scientific), homogenized on ice for 1 min, sonicated (50 W, 3 × 10 s) and centrifuged (14,000 rpm, 30 min). Protein concentration in recovered supernatants was assessed by the Bradford method (Coomassie Plus Protein Assay Reagent—Thermo Scientific). Protein samples (10 µg/lane) were separated by sodium dodecyl sulfate-polyacrylamide gel electrophoresis and transferred (Mini-Protean Tetra Cell and Mini Trans-Blot systems, Bio-Rad, Hercules, CA, USA) to polyvinylidene difluoride membranes (Roche, Mannheim, Germany). Blots were washed, blocked with 5% skim milk and incubated overnight at 4 °C with rabbit primary antibodies. Liver and muscle samples were tested with antibodies against AMPK (1:20,000), phospho-AMPK Thr172 (1:10,000), Acetyl CoA Carboxylase (ACC, 1:2000), its phosphorylated fraction (pACC Ser79, 1:20,000), Akt kinase (1:8000) and phospho-Akt Ser473 (1:2000) (Cell Signaling Technology Inc., Danvers, MA, USA). After being transferred to room temperature and washing, membranes were incubated for 1 h with the peroxidase-conjugated anti-rabbit antibody (Jackson ImmunoResearch Laboratories, 1:20,000). Immunoreactive bands were revealed using the SuperSignal^®^ West Pico PLUS Chemiluminescent Substrate (Thermo Scientific Pierce). The membranes were then stripped (Restore^TM^ PLUS Western Blot Stripping Buffer, Thermo Scientific) and reprobed with the anti-GAPDH or anti-β-actin antibody (1:30,000 and 1:10,000, respectively; Cell Signaling Technology Inc., Danvers, MA, USA). Signals were detected using the Azure c300 Imaging System and quantified by densitometry with the AzureSpot v. 13.1 software (Azure Biosystems, Dublin, CA, USA).

### 2.6. Immunohistochemistry

Pancreatic tissue was collected from animals after decapitation and fixed in Bouin’s solution, then dehydrated in graded alcohols, embedded in paraffin and sectioned. Sections of 5–6 µm in thickness were deparaffinized and re-hydrated. Next, they were boiled in citrate buffer (pH 6, 10 min). After the boiling step sections were cooled to room temperature. Further steps were performed in a humidified chamber at room temperature. The endogenous peroxidase activity was inhibited by incubation of slides in 1% solution of hydrogen peroxide for 30 min. Then slides were washed twice in phosphate -buffered saline—PBS (2 × 10 min). Subsequent incubation in goat serum (1:20 PBS) for 30 min at room temperature was performed to block non-specific antibody binding sites. In the next step, the sections were incubated overnight with the primary antibody at 4 °C (Polyclonal Guinea-pig anti-insulin, 1:200, A0564, purchased from Dako Denmark (Glostrup, Denmark). Subsequently the sections were washed in PBS (3 × 3 min) and incubated with the secondary antibody (anti-Guinea pig, whole molecule, horseradish peroxidase conjugate, 1:200, A7289, Sigma-Aldrich) for 30 min. The sections were washed once again in PBS (3 × 3 min) and developed with the peroxidase substrate—0.5% diaminobenzidine—DAB (Dako, Glostrup, Denmark). After 6 min, sections were washed in water, counterstained with hematoxylin, dehydrated through graded alcohols, cleared in xylene and covered with histofluid (Marienfeld, Lauda-Königshofen, Germany). Control sections involved similarly treated adjacent sections excluding the use of the primary antibody. Preparations were analyzed under an optical microscope (BX40 Olympus, Tokyo, Japan) and scanned using a Panoramic MIDI scanner (3DHISTECH Ltd., Budapest, Hungary). Photos were taken and analyzed with the use of a Mirax Viewer (Zeiss, Jena, Germany).

### 2.7. Liver Histology

Liver samples were collected, stored and fixed similarly to pancreatic tissue. After dehydration in graded alcohols, samples were embedded in paraffin and sectioned, deparaffinized and re-hydrated. Then, they were stained by the hematoxylin and eosin technique (H&E). Preparations were analyzed similarly to pancreatic samples.

### 2.8. Statistical Analysis

The obtained results were expressed as means ± standard error of the mean (SEM) of 10 rats and were evaluated statistically by one-way ANOVA and Tukey’s multiple comparison test using the GraphPad Prism for Windows software (license no. GRA/3802/2015, La Jolla, CA, USA). Differences were considered significant at *p* < 0.05.

## 3. Results

### 3.1. Resveratrol Improves Glucose Tolerance in GK Rats

**Figure 1:**
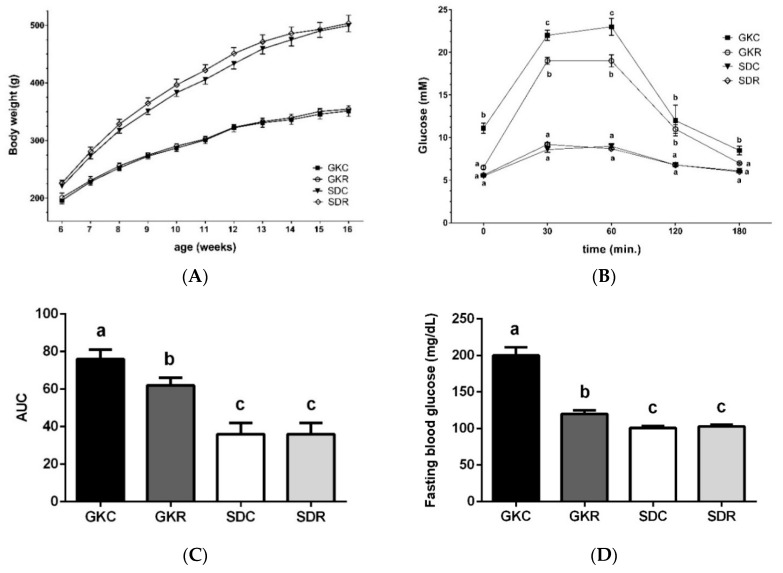

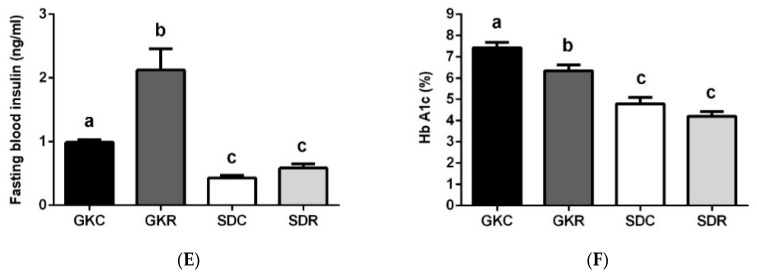
Resveratrol improves glucose tolerance in Goto-Kakizaki (GK) rats. Body weight gains (**A**), time course changes in glycemia after intragastric glucose load (**B**), area under the curve for blood glucose calculated during glucose tolerance test (**C**), fasting blood glucose (**D**), fasting blood insulin (**E**), and glycated hemoglobin (HbA1c) levels in blood of non-fasted rats (**F**). GKC—Goto-Kakizaki control rats, GKR—Goto-Kakizaki resveratrol-treated rats, SDC—Sprague–Dawley control rats, SDR—Sprague–Dawley resveratrol-treated rats. Results are expressed as the means ± standard error of the mean (SEM) from 10 rats in each group. All parameters, except for HbA1c, were measured in fasted rats after eight weeks of the experiment, whereas HbA1c was determined in non-fasted animals after 10 weeks of the study. Values with different letters are significantly different at *p* < 0.05.

As expected, body weight gains were significantly lower in non-obese GK rats compared with age- and sex-matched control animals. Resveratrol had no significant influence on body weight gains in any of the investigated groups (Figure 1A). Results of the glucose tolerance test revealed that after glucose loading glycemia was highly increased in diabetic animals compared with Sprague–Dawley (SD) rats. This increase was, however, significantly reduced in GKR rats (Figure 1B). The difference between the groups was also evident when the area under the curve (AUC) was compared. It was shown that AUC was twice as large in the GKC group compared with non-diabetic animals. However, in the GKR group, AUC was significantly diminished (Figure 1C). It was also demonstrated that diabetic rats display a clear-cut fasting hyperglycemia. Importantly, in the group of rats treated with the tested compound their fasting blood glucose level was decreased nearly to the values found in control animals (Figure 1D). In the present study, fasting blood insulin levels were elevated in GK rats compared with the non-diabetic control. Resveratrol was found to induce an additional rise in insulin concentration (Figure 1E). Moreover, it was revealed that diabetic rats had higher blood levels of HbA1c. However, consistent with results addressing beneficial effects of resveratrol on concentrations of blood glucose, we also showed that treatment of GK rats with this compound was associated with a drop in blood HbA1c (Figure 1F).

### 3.2. Effects of Resveratrol on Blood Adipokine Levels

**Figure 2:**
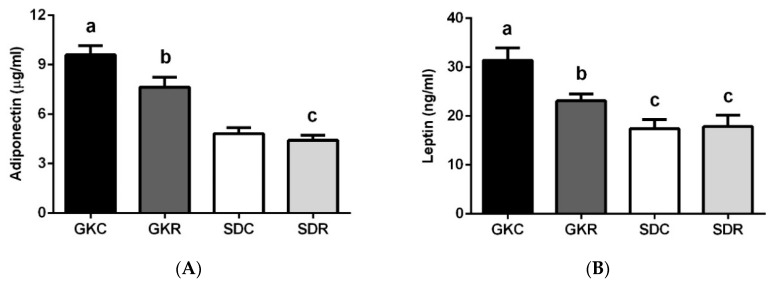

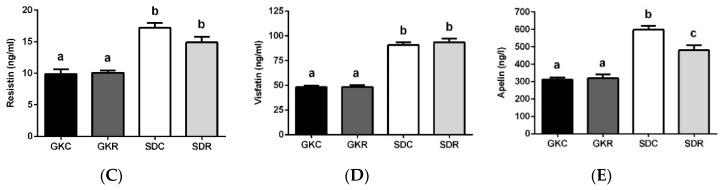
Effects of resveratrol on blood adipokine levels in GK and Sprague–Dawley (SD) rats. Adiponectin (**A**), leptin (**B**), resistin (**C**), visfatin (**D**), apelin (**E**). GKC—Goto-Kakizaki control rats, GKR—Goto-Kakizaki resveratrol-treated rats, SDC—Sprague–Dawley control rats, SDR—Sprague–Dawley resveratrol-treated rats. Results are expressed as the means ± SEM from 10 rats in each group. Values with different letters are significantly different at *p* < 0.05.

In the present study, concentrations of blood adiponectin were markedly higher in diabetic rats compared with control animals. However, as a result of resveratrol treatment a significant reduction of blood adiponectin levels was shown (Figure 2A). Similarly to adiponectin, GK rats also had augmented blood leptin levels. In this case, the tested compound appeared to also be effective, since hyperleptinemia was reduced in the GKR group (Figure 2B). Although resveratrol did not reduce blood adiponectin or leptin levels to values shown in SD rats, its influence in both cases was significant. In our present study concentrations of blood resistin, visfatin and apelin were significantly diminished in diabetic rats compared with control animals. It was shown that resveratrol treatment had no influence on these adipokines (Figure 2C–E). Moreover, it was observed that resveratrol diminished blood apelin levels in SD rats.

### 3.3. Effects of Resveratrol on Skeletal Muscle Lipids, Expression and Phosphorylation of AMPK, ACC and Akt in Muscle Tissue

**Figure 3:**
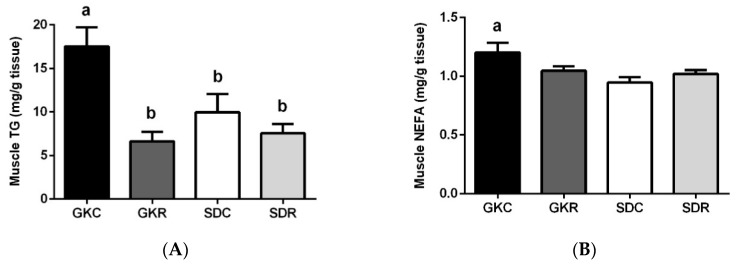

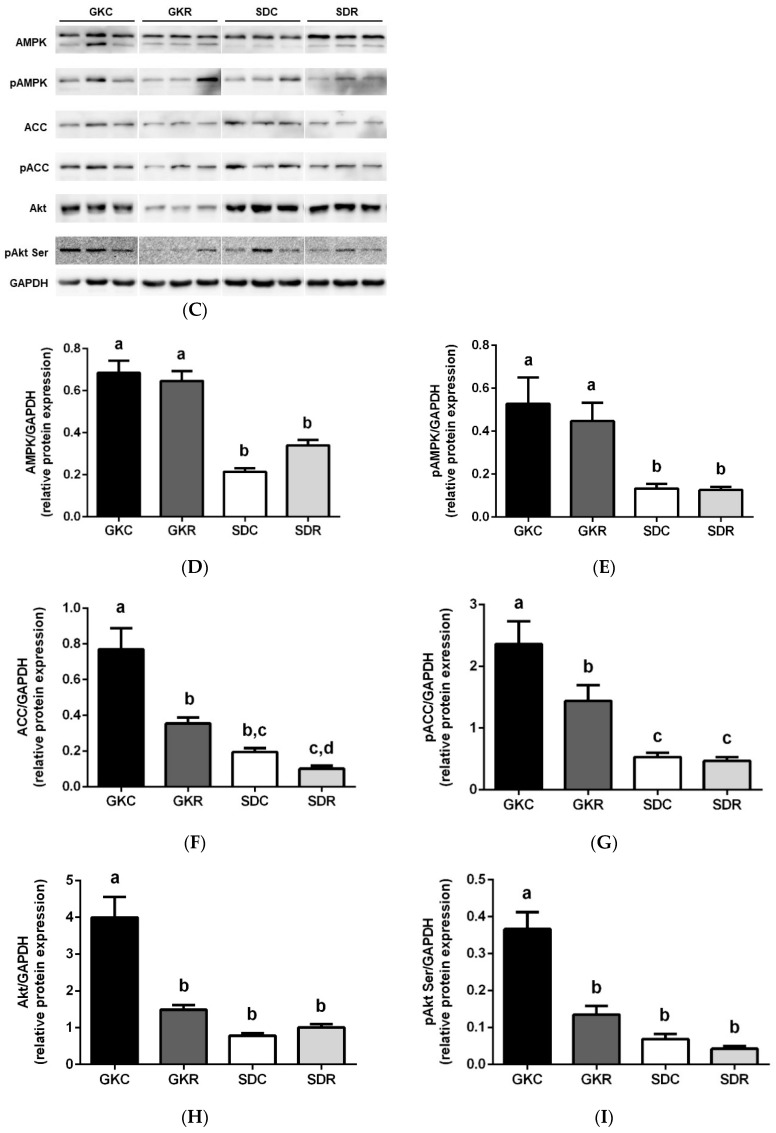
Effects of resveratrol on skeletal muscle lipids, and also expression and phosphorylation of activated protein kinase (AMPK), acetyl-CoA carboxylase (ACC) and protein kinase B (Akt) in muscle tissue of GK and SD rats. Triglycerides (**A**), non-esterified fatty acids (**B**). Western blot immunodetection with antibodies to the indicated proteins, GAPDH expression used as loading control (**C**). Protein levels relative to GAPDH quantified by densitometry: Total AMPK (**D**) and phosphorylation (pAMPK) (**E**), total ACC (**F**) and phosphorylated fraction (pACC) (**G**), total Akt (**H**) and pAkt (**I**). Results are representative of at least two independent experiments. GKC—Goto-Kakizaki control rats, GKR—Goto-Kakizaki resveratrol-treated rats, SDC—Sprague–Dawley control rats, SDR—Sprague–Dawley resveratrol-treated rats. Results are expressed as the means ± SEM from 10 rats in each group. Values with different letters are significantly different at *p* < 0.05.

Our study revealed that contents of TG and NEFA were significantly increased in the skeletal muscle of GK rats compared with control animals. The difference between SD and GK rats was particularly large in the case of TG. Importantly, increased accumulation of TG in the skeletal muscle of GK rats was totally prevented as a result of resveratrol therapy (Figure 3A). It was also shown that NEFA content was slightly reduced in GKR (Figure 3B).

Western blot analysis revealed the impact of resveratrol on the level and activity of AMPK, ACC and Akt enzymes (Figure 3C–I). We demonstrated that expression of AMPK as well as its phosphorylation (pAMPK) in the skeletal muscle of GK rats was markedly augmented compared with SD rats. In the present study, resveratrol failed to affect expression levels of AMPK and pAMPK (Figure 3D,E). Apart from up-regulation of AMPK, GK rats were also characterized by an exaggerated expression and phosphorylation of ACC in the muscle tissue compared with data shown in SD rats. However, in the GKR group, the ACC expression level along with its phosphorylation (pACC) was found to be markedly reduced. The influence of resveratrol was particularly evident in the case of the active form of the enzyme (ACC) (Figure 3F). We next studied the expression of Akt and pAkt in the skeletal muscle of control and diabetic rats. It was shown that expression levels of this protein as well as its phosphorylation were substantially augmented in insulin-resistant GK rats. However, resveratrol therapy was associated with a substantial decline in the expression of Akt and pAkt. In this group of diabetic rats, the expression level of this signaling protein was close to the values found in the control group (Figure 3H,I).

### 3.4. Effects of Resveratrol on Liver Lipids, Expression and Phosphorylation of AMPK, ACC and Akt in Liver Tissue

**Figure 4:**
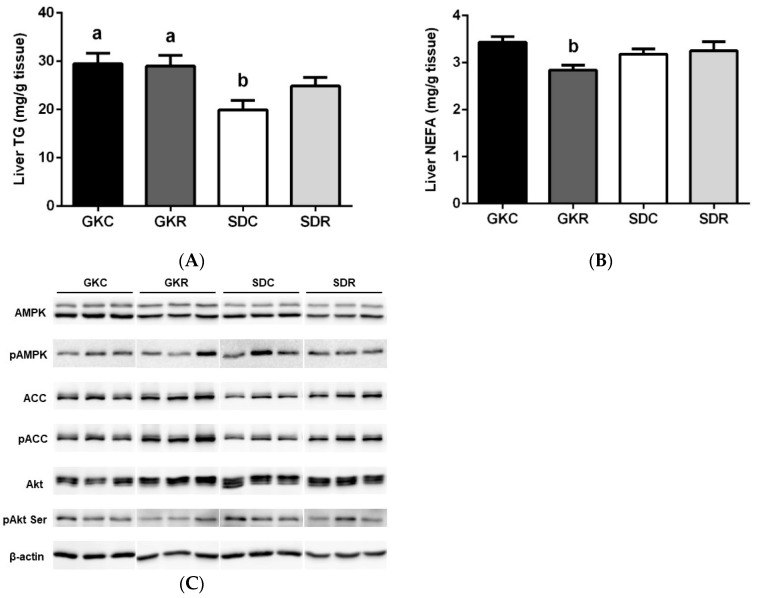

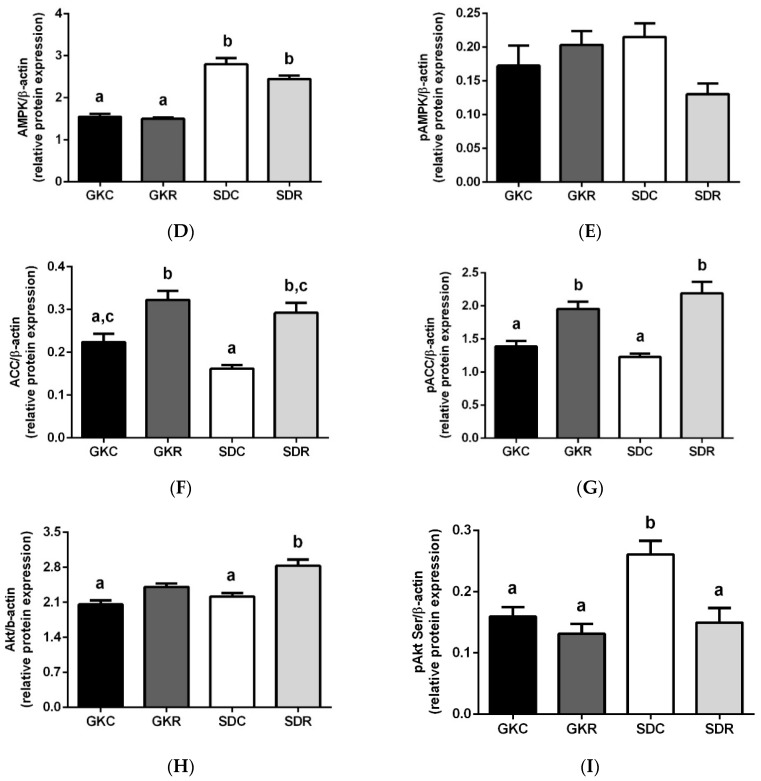
Effects of resveratrol on liver lipids, and also expression and phosphorylation of AMPK, ACC and Akt in liver tissue of GK and SD rats. Triglycerides (**A**), non-esterified fatty acids (**B**). Western blot immunodetection with antibodies to the indicated proteins, β-actin expression used as loading control (**C**). Protein levels relative to β-actin quantified by densitometry: Total AMPK (**D**) and pAMPK (**E**), total ACC (**F**) and pACC (**G**), total Akt (**H**) and pAkt (**I**). Results are representative of at least two independent experiments. GKC—Goto-Kakizaki control rats, GKR—Goto-Kakizaki resveratrol-treated rats, SDC—Sprague–Dawley control rats, SDR—Sprague–Dawley resveratrol-treated rats. Results are expressed as the means ± SEM from 10 rats in each group. Values with different letters are significantly different at *p* < 0.05.

Compared with skeletal muscle, liver lipids were less affected in diabetic rats. It was shown that in GK rats, liver TG was moderately increased, with no influence of resveratrol found (Figure 4A). Moreover, NEFA contents were comparable in each group of animals, whereas resveratrol significantly diminished NEFA in GK rats (Figure 4B).

To fully compare the effects of resveratrol treatment in the skeletal muscle and liver, Western blot detection of the same enzymes was performed (Figure 4C–I). Liver AMPK was down-regulated in GK rats; however, its expression was not influenced by resveratrol treatment (Figure 4D). Moreover, phosphorylation of AMPK (the active form of this enzyme) did not significantly differ between the studied groups (Figure 4E).

It was also revealed that liver ACC and pACC levels were comparable in GK and SD rats. In our present study, resveratrol significantly increased the expression and phosphorylation of this enzyme in both groups (Figure 4F,G). Moreover, expression of liver Akt was found to be similar in the control and in diabetic rats, whereas the level of pAkt was significantly lower in GK rats and resveratrol failed to affect phosphorylation of this signaling molecule (Figure 4H,I). It was also shown, that in SD rats, resveratrol increased the expression of Akt and decreased the expression of pAkt (Figure 4H,I).

### 3.5. Effects of Resveratrol on Morphology of Pancreatic Islets and Liver Histology

**Figure 5:**
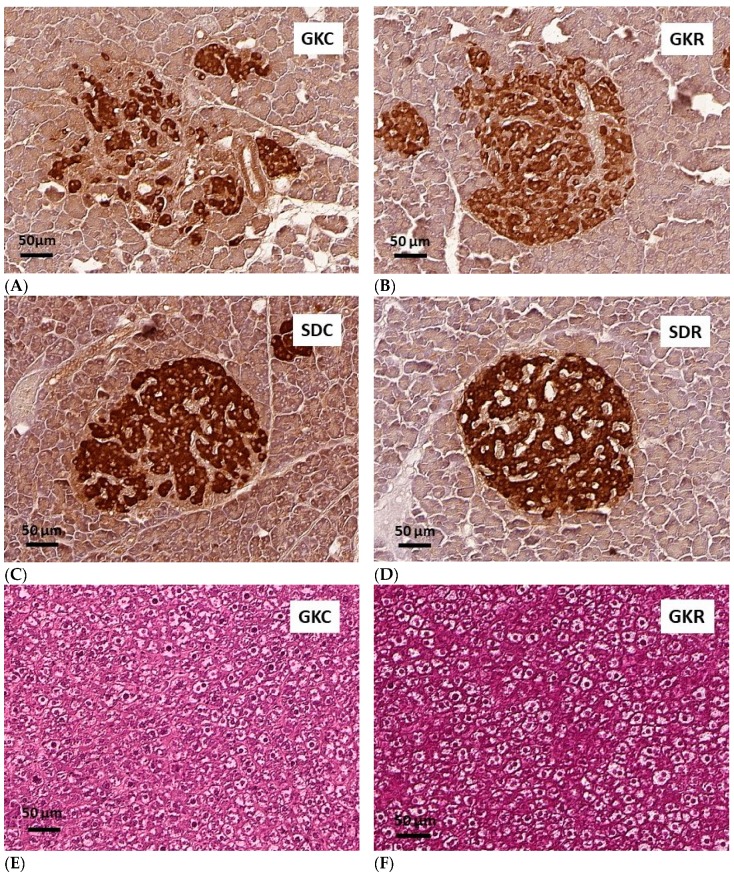

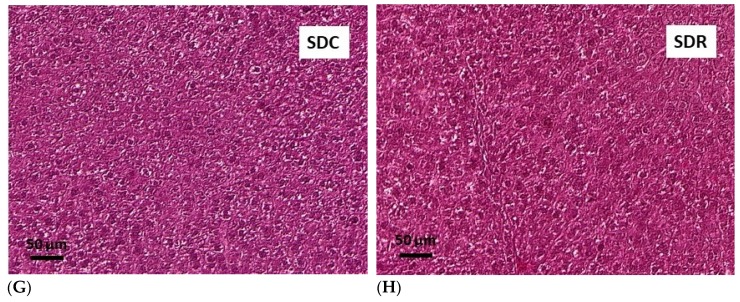
Effects of resveratrol on morphology of pancreatic islets and liver histology in GK and SD rats. GKC—Goto-Kakizaki control rats, GKR—Goto-Kakizaki resveratrol-treated rats, SDC—Sprague–Dawley control rats, SDR—Sprague–Dawley resveratrol-treated rats. The representative images of immunostaining for insulin of pancreatic islets counterstained with hematoxylin (**A–D**), the representative images of H&E staining of hepatic tissue (**E–H**). Magnifications shown by scale bars.

To compare the morphology of pancreatic islets in each group of rats, the pancreatic tissue was sampled and immunostaining for insulin was performed. This allowed us to reveal substantial differences between islets derived from control and diabetic rats. In non-diabetic SD rats, irrespective of resveratrol treatment, pancreatic islets displayed normal structure. However, GK rats have two populations of pancreatic islets, namely small islets with a normal architecture (not shown) and large islets characterized by an irregular structure and connective tissue (Figure 5A). Resveratrol therapy was found to partially improve morphology of large islets in GK rats. It was shown that in the group of diabetic animals treated with this compound, pancreatic islets were much less irregular compared with islets of vehicle-treated GK rats (Figure 5B). These results allow us to conclude that resveratrol improves islet integrity in GK rats.

Histological examination of liver tissue using the hematoxylin and eosin technique showed some visible differences between livers of GK and SD rats; however, resveratrol was found to have no influence here (Figure 5E–H).

## 4. Discussion

Fasting hyperglycemia, shown in the present study in insulin-resistant GK rats, is one of the key hallmarks of type 2 diabetes and is strongly associated with numerous diabetes complications. Therefore, the blood-glucose lowering action limits progression of the disease and it also is essential in management of diabetes. In our study, resveratrol effectively decreased fasting blood glucose and hemoglobin A1c levels in GK rats. Moreover, the glucose tolerance test revealed increased glucose clearance in these animals following resveratrol treatment. These findings indicate that the tested compound is capable of improving glycemic control in GK rats. The blood-glucose lowering properties of resveratrol are relatively well established in animal models with diet-induced insulin resistance. The beneficial effect of resveratrol on glycemic control found in GK rats appears to be largely due to its influence on skeletal muscle lipid accumulation. It was shown that in the skeletal muscle of these rats, contents of TG and NEFA were significantly increased compared with the control. The difference was particularly evident in the case of TG. A similar rise in TG content in the skeletal muscle of GK rats was reported previously; however, the mechanism underlying this disorder has not been elucidated to date [24,25]. In our study, resveratrol therapy totally prevented TG accumulation, while it also partially reduced NEFA content. This is a very important effect, given that under physiological conditions, skeletal muscles absorb considerable amounts of circulating glucose and are the major sites of insulin-induced glucose uptake. However, a rise in intramyocellular lipids is associated with impaired insulin action and reduced glucose uptake by muscle cells, which in turn largely contributes to hyperglycemia [26,27,28]. It was previously shown that in GK rats, insulin-induced glucose uptake by the skeletal muscle was indeed lower than in control animals [29,30]. Moreover, insulin resistance in the skeletal muscle is thought to be the primary defect in type 2 diabetes [31], whereas reduced lipid accumulation in this tissue, with a concomitant improvement of intramuscular glucose transport, positively affects whole-body glucose homeostasis [32,33].

In order to suggest the underlying mechanism, through which resveratrol reduces lipid content in the skeletal muscle of GK rats, effects of this compound on the expression of genes encoding the relevant enzymes and signaling molecules has been studied. The beneficial action of resveratrol in animal models often covers changes in the expression/activity of AMP-activated protein kinase (AMPK). This enzyme, being an important intracellular energy sensor, is implicated in the regulation of metabolic pathways and plays a role in the maintenance of energy homeostasis [34]. One of the intracellular AMPK targets is acetyl-CoA carboxylase (ACC), which catalyzes conversion of acetyl-CoA to malonyl-CoA. Phosphorylation of ACC by pAMPK represses this enzyme, which reduces formation of malonyl-CoA and promotes β-oxidation [34]. The action of resveratrol via AMPK is relatively well established in animal models with diet-induced obesity and insulin resistance. AMPK is usually down-regulated, whereas resveratrol prevents this effect. However, the response to resveratrol treatment may vary and it may also be tissue specific [6,12,35]. In the present study protein expression of AMPK and its phosphorylation (pAMPK) in the muscle tissue of GK rats was augmented compared with the control group, with no significant effect of resveratrol observed in this respect. Moreover, it was shown that the expression of ACC and pACC was also exaggerated in the skeletal muscle derived from diabetic animals. Importantly, in this case the tested compound appeared to be effective, since in GK rats subjected to resveratrol therapy, the expression levels of ACC (active form) and pACC were substantially reduced. Our results strongly suggest AMPK-independent effects of resveratrol on the expression and phosphorylation of ACC. This is highly probable, given that AMPK is not the sole regulator of ACC. Moreover, the AMPK-independent influence of resveratrol on skeletal muscle ACC was demonstrated in other model of diabetes [36].

Up-regulation of the ACC system, revealed in our study in diabetic rats, is known to be associated with increased intracellular levels of malonyl-CoA. Such an effect was previously found in the skeletal muscle of humans with type 2 diabetes, in which an excessive activity of ACC was accompanied by elevated amounts of malonyl-CoA [37], as well as the skeletal muscle of GK rats [38]. Given that a rise in intracellular malonyl-CoA reduces β-oxidation and simultaneously favors lipid deposition [39,40], the capability of resveratrol to downregulate ACC largely explains the positive influence of this compound on skeletal muscle lipid content and the associated blood-glucose lowering effects.

Pathogenesis of skeletal muscle insulin resistance in GK rats is poorly elucidated. It is, however, known that the action of some molecules of the insulin signaling pathway is dysregulated [19,41,42,43]. In the present study the effects of resveratrol on the expression and phosphorylation of one of these molecules, namely Akt (protein kinase B), were explored. Under physiological conditions, pAkt phosphorylates GSK-3β (glycogen synthase kinase 3 beta) to transmit the insulin signal [44]. Importantly, Akt is involved not only in insulin action, but is also implicated in the adiponectin signaling pathway [45]. In our study the expression of Akt protein and its phosphorylation level (pAkt) in the skeletal muscle of insulin-resistant GK rats were excessive compared with control animals. This is in line with previous outcomes showing that in the skeletal muscle of GK rats pAkt was unable to phosphorylate GSK-3β, because both signaling proteins were already highly phosphorylated, which was strongly associated with insulin resistance [19,41,42,43]. However, we have shown that resveratrol treatment restored both the expression and phosphorylation of Akt to the level found in non-diabetic animals. Given that the high level of pAkt in skeletal muscle of GK rats has been reported to largely contribute to insulin resistance [19,41,42,43], this effect seems to be highly relevant in the mechanism of resveratrol action, especially in the context of intramuscular glucose transport. It is known that among two different mechanisms of glucose uptake by the skeletal muscle, i.e., the Akt and AMPK signaling pathways, the former is insulin-dependent and is responsible for a major part of transported glucose, whereas the AMPK-mediated pathway is linked to physical activity. These findings strongly suggest that resveratrol may promote glucose uptake without affecting skeletal muscle AMPK. Moreover, our results showing normalization of ACC and Akt under conditions when both these proteins were upregulated, seem to be very interesting and indicate new possibilities of resveratrol action.

Apart from skeletal muscle, liver lipid metabolism is also known to be dysregulated in GK rats. However, hepatic fat accumulation may be unchanged or moderately augmented, since both fatty acid synthesis and oxidation are upregulated [19,46]. Consistent with this notion, it was shown that liver TG content was increased in GK rats, but this increase was lower compared with the muscle tissue, at no significant effect of resveratrol observed. Moreover, changes in the expression levels of analyzed proteins in the liver tissue were lower than in skeletal muscle. Hepatic expression of pAMPK (the active form) did not markedly differ between the investigated groups. Expression of pACC and ACC in GK rats was similar to that found in control animals, with resveratrol increasing the expression of this enzyme. It was also shown that the level of pAkt in GK rats was lower than in the control group, although resveratrol failed to affect its phosphorylation. Moreover, the expression of Akt was similar in each group. Comparing pathological changes developing in skeletal muscle and livers of diabetic rats it can be presumed that the liver is less affected. This is in line with findings of the previous studies [19]. Moreover, our results indicate that the action of resveratrol in GK rats is tissue-specific. Similar effects of resveratrol were observed in some other animal models with insulin resistance [35].

In GK rats, hyperglycemia develops as a result of both impaired insulin secretion and action [18]. A gradual failure of pancreatic β-cells is associated with numerous defects, including decreased glucose transport and metabolism, islet fibrosis and disintegration, reduced β-cell mass and impaired insulin-secretory response [20]. These defects are manifested by age-related changes in blood insulin levels. In younger rats, blood insulin level is elevated (due to insulin resistance) compared with the non-diabetic control, to finally decline with ageing (as a result of progressive β-cell failure) below physiological values [20,39,47]. In the present study, resveratrol therapy was associated with increased fasting blood insulin levels in GK rats with a concomitant decrease of hyperglycemia. This suggests that the tested compound improved the insulin-secretory capacity of β-cells in these animals. Given that chronically elevated blood glucose levels largely contribute to the failure of β-cells [18,20], this favorable influence of resveratrol may be partially ascribed to increased glucose uptake by the skeletal muscle and the resulting blood-glucose lowering effects. Histological examination of pancreatic tissue revealed substantial differences between islets of control and diabetic animals. GK rats typically have two populations of pancreatic islets, i.e., large islets with impaired integrity and pronounced fibrosis, and small islets of normal architecture [20]. These structural and functional defects may be alleviated by some anti-diabetic drugs [18,21]. In our study, two populations of pancreatic islets were indeed seen in diabetic rats. Large islets in GK rats displayed a characteristic irregular structure; however, resveratrol treatment clearly improved islet integrity in this group of animals. These outcomes show a positive influence of the tested compound on islet structure in diabetic rats.

Apart from insulin, we also focused on adipokines largely implicated in glucose homeostasis and having other relevant regulatory roles. One of them is adiponectin, which participates in the control of numerous intracellular processes acting mostly in concert with insulin [48]. However, chronically elevated blood adiponectin levels are associated with adiponectin and insulin resistance [49]. In the present study, consistent with previous data [47,50], circulating adiponectin levels in GK rats were significantly elevated, whereas resveratrol therapy reduced blood levels of this hormone. This effect may be expected to contribute to the improvement in the action of adiponectin on target tissues, especially the skeletal muscle. This is a relevant finding, given that adiponectin promotes β-oxidation in skeletal muscle and thereby diminishes accumulation of lipids in this tissue. In contrast, insulin and adiponectin resistance impairs the ability of this adipokine to stimulate β-oxidation [51]. Blood leptin levels were also increased in GK rats. White fat tissue is the main source of this hormone; however, in GK rats blood leptin levels were found to be elevated in spite of reduced adiposity [47,52,53]. Leptin regulates, among other things, feeding behavior, energy expenditure and glucose homeostasis. However, chronic hyperleptinemia leads to leptin resistance and is associated with insulin resistance and hyperglycemia [54,55]. Symptoms of leptin resistance in the hypothalamus and peripheral tissues have been indeed revealed in GK rats [52,53]. In the present study, the treatment of diabetic rats with resveratrol diminished blood leptin levels, which may improve the action of this hormone on target tissues.

Experimental data show that in rodent models of diet-induced obesity, or in the case of genetic forms of obesity, blood resistin levels are elevated. This rise is well established to contribute to the onset of insulin resistance in liver, skeletal muscle and adipose tissue. However, the link between resistin and insulin resistance in humans has not been proven [56]. In our study, the concentration of circulating resistin in diabetic animals was lower compared with the control, which suggests that this hormone is not implicated in the development of insulin resistance in GK rats. Similarly to resistin, blood apelin and visfatin levels were also decreased in diabetic rats, with no effect of resveratrol observed.

In animals with obesity and insulin resistance induced by high-calorie diet, beneficial effects of resveratrol are usually associated with reduction in body weight and adiposity. In the present study this compound was shown to ameliorate diabetes in GK rats without affecting body weight. This indicates the effectiveness of resveratrol in the non-obese model of type 2 diabetes, while it also demonstrates that a decrease in body weight is not a prerequisite for its action.

Rodent studies with diet-induced insulin resistance show that effective doses of resveratrol vary greatly. Our results demonstrate that this compound is capable of inducing favorable changes in GK rats when administered at a relatively low dose, which is not accompanied by harmful effects.

Our study has some limitations. We used SD rats as the control. They are less commonly used than Wistar rats; however, several parameters related to metabolism in SD and Wistar rats are similar [57]. Moreover, both GK rats and SD rats are used in studies on new anti-diabetic compounds [57,58,59,60] and anti-diabetic therapies [61,62]. Another limitation was connected with using only male rats. It would be interesting to compare potential sex differences addressing resveratrol action in GK rats. However, such an approach within one study would cause serious technical problems.

## 5. Conclusions

Our study shows, for the first time, the efficacy of resveratrol in the GK rat, a non-obese model of type 2 diabetes determined genetically. The main finding is that resveratrol therapy prevents skeletal muscle lipid accumulation, which is largely associated with normalization of ACC and Akt levels. This is particularly relevant, considering the link between skeletal muscle lipids and pathogenesis of type 2 diabetes. Moreover, resveratrol was demonstrated to improve glycemic control. Given a different etiology of diabetes in the model used in the present study, our results point at new possibilities for resveratrol action.
